# α-catenin SUMOylation increases IκBα stability and inhibits breast cancer progression

**DOI:** 10.1038/s41389-018-0037-7

**Published:** 2018-03-13

**Authors:** Huan Chen, Zhaowei Xu, Xiahui Li, Yangyang Yang, Bowen Li, Yanan Li, Kangkai Xia, Jian Wang, Shujing Li, Miao Wang, Huijian Wu

**Affiliations:** 1 School of Life Science and Biotechnology, Dalian University of Technology, Dalian, China; 2 School of Life Science and Medicine, Dalian University of Technology, Panjin, China

## Abstract

α-catenin has been demonstrated to suppress several different types of cancers. Here we demonstrate that α-catenin is modified by SUMO protein, which covalently binds α-catenin at the carboxy terminus at lysine 870. Substitution of lysine 870 with arginine completely abolishes α-catenin SUMOylation. This modification can be removed by SENP1. However, α-catenin SUMOylation does not affect its stability and subcellular localization. In addition, we observed that the SUMOylation-deficient α-catenin mutant has a reduced interaction with IκBα which prevents subsequent ubiquitination of IκBα, and therefore a reduced suppression of expression of the NF-κB target genes *TNF-α*, *IL-8*, *VEGF*, and *uPA*. In addition, the α-catenin SUMOylation mutant shows impaired suppression of tumor growth. These results demonstrate that SUMOylation at lysine 870 of α-catenin plays a key role in the suppression of the NF-κB pathway, which inhibits breast cancer tumor growth and migration.

## Introduction

The tumor-suppressor gene *CTNNA1* (encoding α-catenin) is one of the most frequently deleted^1,2^ or mutated^3^ genes in cancer. α-catenin is an essential protein in adherens junctions, which are critical for maintaining intercellular adhesion and cellular polarity^4,5^. The tumor-suppressor activities of α-catenin involve following signaling pathways. An siRNA screen suggested that the YAP1 may be a downstream effector of α-catenin^6^, which inhibits the transcriptional activity of YAP1 by promoting its cytoplasmic sequestration^7^. α-catenin is frequently downregulated in human skin cancer^8^, and an α-catenin-knockout skin squamous cell carcinoma mouse model showed upregulation of many NF-κB downstream genes^9^. α-catenin suppresses p50/p65 nuclear localization by binding to IκBα and increasing its protein stability in breast cancer^10^. Moreover, α-catenin directly interacts and promotes β-catenin degradation^11^.

SUMOylation is a post-translational modification where SUMO covalently binds to target proteins^12^. SUMOylation regulates numerous cellular stress responses including DNA repair, transcriptional regulation, and RNA metabolism^12,13^. Like with ubiquitination, SUMOylation involves several steps and three classes of enzymes including E1 enzyme SAE1/SAE2, only E2 enzyme Ubc9 and diverse E3 enzymes which accelerate the SUMOylation rate of final step^14^. SUMOylation is a dynamic process which regulates substrate functions including subcellular distribution, protein stability and control of protein interactions^15,16^. Numerous studies indicate that NF-κB pathway, such as stabilizing IκBα^17^, activation of the IκB kinase NEMO^18^, and promoting p50 maturation^19^, is regulated by SUMOylation. Given that α-catenin can interact with Ubc9^20^ indicates that α-catenin is a target of SUMO. A computer analysis of the α-catenin protein sequence reveals two conserved SUMOylation sites: lysine 86 and 870. In this article, we demonstrate that α-catenin is SUMOylated at lysine 870 in vivo. Mutating K870 to arginine reduces the binding of α-catenin to IκBα and negatively regulates NF-κB target gene expression, therefore inhibiting α-catenin tumor-suppressor activity in breast cancer. Collectively, our findings suggest that α-catenin SUMOylation is of great importance in regulating α-catenin tumor-suppressor activity.

## Results

### α-catenin is modified by SUMO

**Fig. 1:**
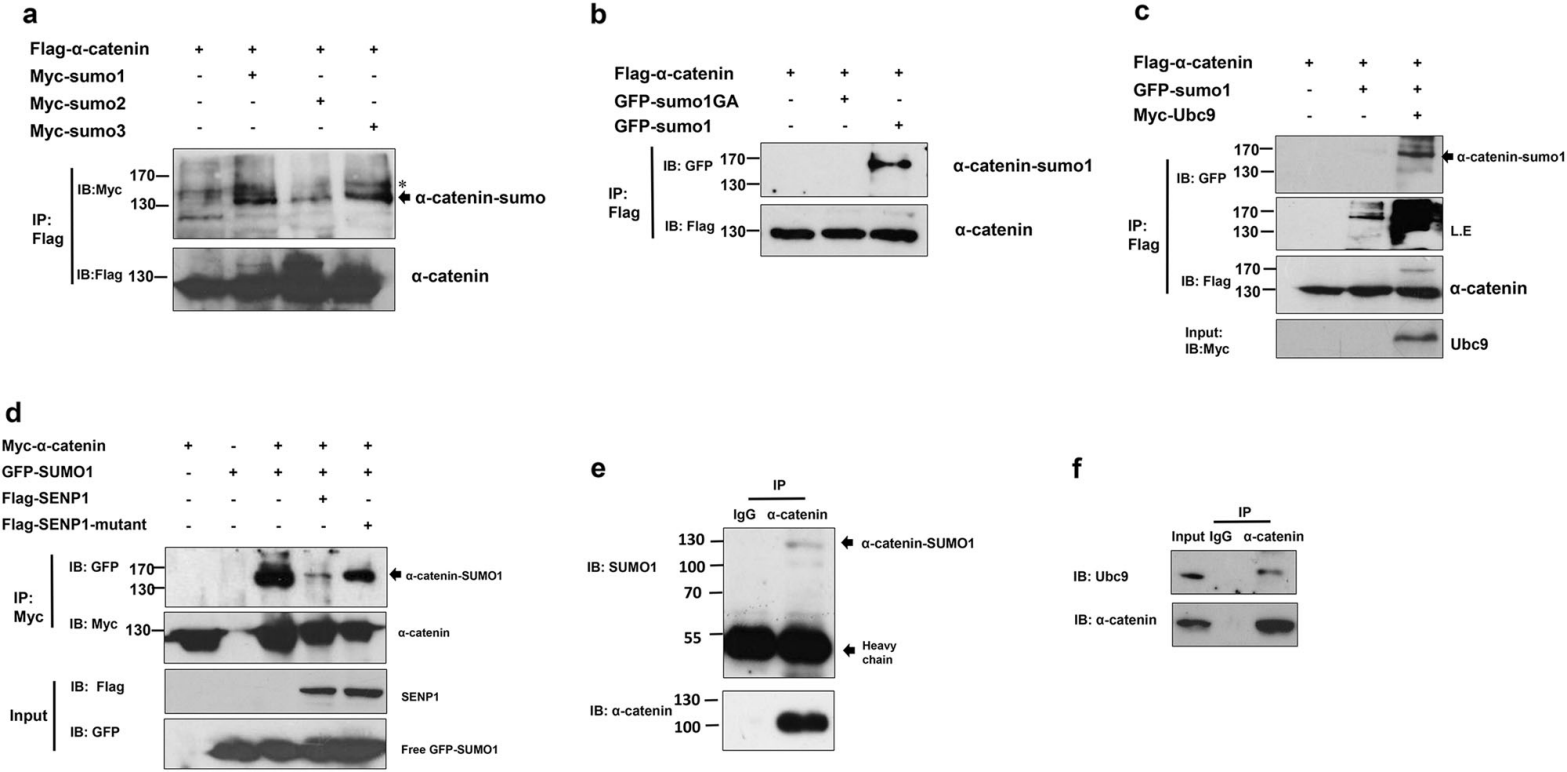
α-catenin is Modified by SUMO. **a** HEK293T cells were co-transfected with relevant plasmids, cells were collected at 48 h after transfection and immunoprecipitated with anti-Flag gel (Bimake, South Loop West, Suite 175, Houston, B23101). “*” non-specific bands. **b** HEK293T cells co-transfected with GFP-SUMO1 or its SUMOylation mutant and Flag-α-catenin. Cell extracts were inmmunoprecipitated with Flag gel described in **a**. **c** HEK293T cells were transfected with indicated plasmids and treated with the same protocol in **a**. **d** HEK293T cells were transfected relevant plasmids and the cell lysates were immunoprecipitated with Myc-tag antibody. **e** Endegenous α-catenin-SUMO1 were purified with denaturing condition as described in methods. **f** HEK293T cell lysates were immunoprecipitated with anti-α-catenin antibody or control IgG followed by western blot with anti-Ubc9 antibody

To determine whether α-catenin is modified by SUMO, Exogenous α-catenin SUMOylation were detected in HEK293T cells co-transfected with Flag-labeled α-catenin together with Myc-labeled SUMO1, SUMO2, SUMO3 or empty vector. Flag-tagged proteins were purified in NEM-TNE lysis buffer using Flag gel. We observed Flag-α-catenin-SUMO bands only when cells were co-transfected with three types of SUMO protein (Fig. 1a), indicating that α-catenin is substrate of SUMO protein. Since SUMO1 modifies target proteins via its glycine 97, and mutating G97 to arginine is enough to destroy modification function^21^. To further confirm this, HEK293T cells were transfected with Flag-α-catenin together with WT SUMO1 or its mutant SUMO1-G97A and analyzed the SUMOylated proteins. We observed α-catenin was only modified by WT SUMO1 but not SUMO-G97A (Fig. 1b). In addition, the abundance of SUMOylation can be significantly enhanced by Ubc9, a SUMO E2 conjugating enzyme^22^. Cells co-transfected with Ubc9 and SUMO1 showed significantly higher amounts of the α-catenin-SUMO1 band (Fig. 1c). Moreover, we observed that SUMO1 could be removed from α-catenin by WT SENP1 instead of catalytic mutant SENP1 (SENP1m) (Fig. 1d). We next performed SUMOlation assays in HEK293T cells to detect endogenous α-catenin-SUMO1 in vivo, α-catenin was immunoprecipitated with α-catenin antibody and we subsequently observed SUMOylated α-catenin (Fig. 1e). In addition, endogenous α-catenin co-immunoprecipitated with Ubc9 (Fig. 1f). Together, α-catenin is modified by SUMO protein.

### SUMO modification of α-catenin at lysine 870

**Fig. 2:**
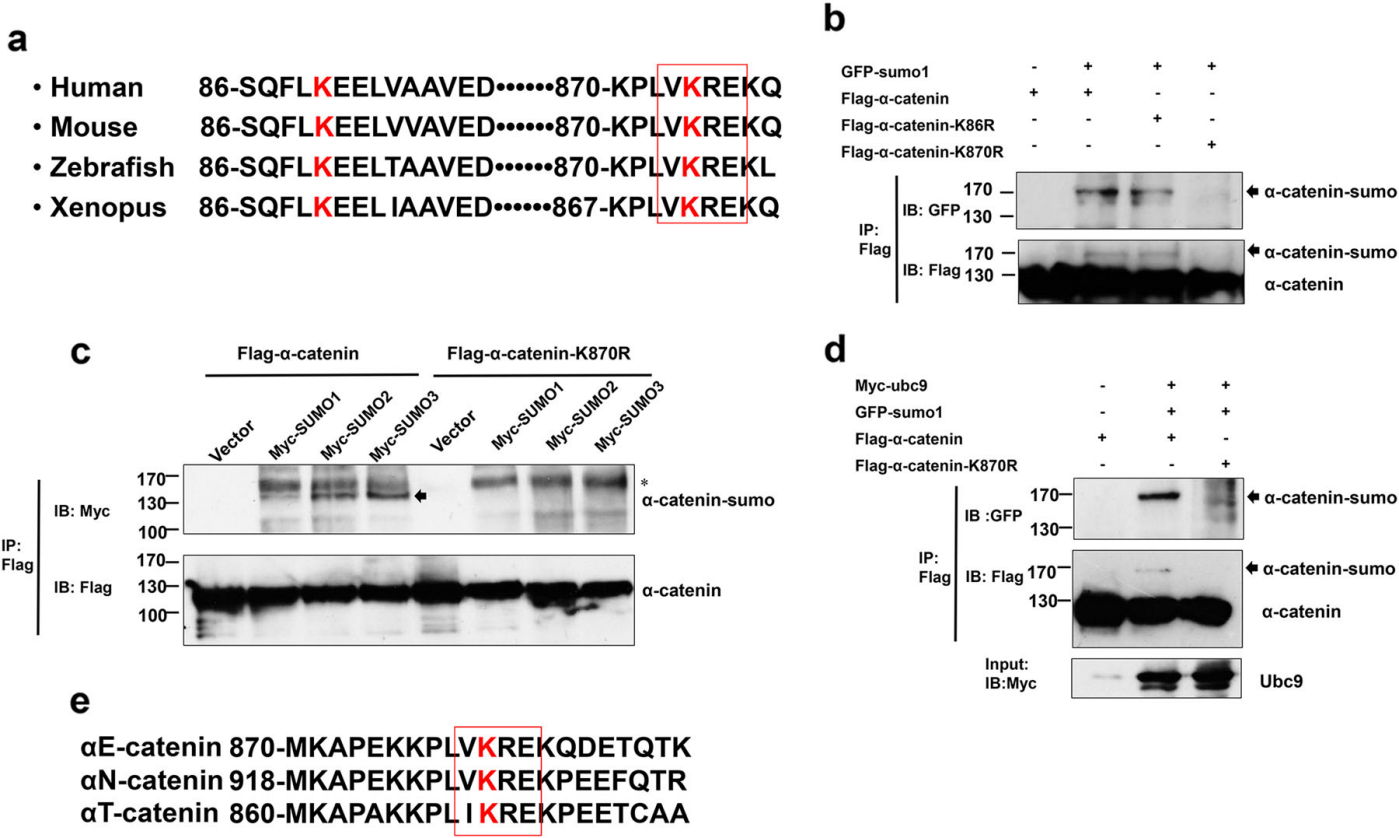
SUMO modification of α-catenin at lysine 870. **a** Sequences and locations of putative α-catenin SUMO modification sites in the human, mouse, zebrafish and xenopus. **b** HEK293T cells were transfected with indicated plasmids, and cell lysates were purified with flag gel. Purified cell exctracts were detected using western blot. **c**, **d** HEK293T cells were transfected with relevant plasmids and tested using the same methods as **b**. “*” non-specific bands. **e** SUMOylation sites in CTNNA1, CTNNA2 and CTNNA3

A computer analysis of the α-catenin protein sequence revealed two classical SUMOylation consensus motifs including lysine 86 and 870. In addition, two potential SUMOlation motif are conserved in diverse species (homo sapiens, mouse, zebrafish, xenopus) (Fig. 2a). We constructed single point mutations in the putative lysine residues of α-catenin and analyzed SUMO conjugation. SUMOylated α-catenin band was totally lost when cells were co-transfected with α-catenin K870R (Fig. 2b). Similarly, SUMO2 and SUMO3 conjugation to α-catenin was nearly dissolved with the K870R α-catenin mutation (Fig. 2c). These results point to the lysine residue at position 870 as a SUMO-acceptor site in α-catenin. To verify this information, Ubc9 was used to enhance the SUMOylation level of WT α-catenin and the K870R α-catenin mutant. As expected, α-catenin SUMOylation was completely abolished in the K870R α-catenin mutant, even when cells were co-transfected with Ubc9 (Fig. 2d). In mammals, there are three α-catenin proteins including αE-catenin, αN-catenin and αT-catenin, which are encoded by *CTNNA1*, *CTNNA2* and *CTNNA3*, respectively^23^. Surprisingly, we also observed similar SUMOylation sites in αN-catenin and αT-catenin (Fig. 2e). These data indicate that lysine residues at position 870 of α-catenin is implicated in the conjugating to SUMO.

### SUMOylation does not affect stability of α-catenin and cellular localization

**Fig. 3:**
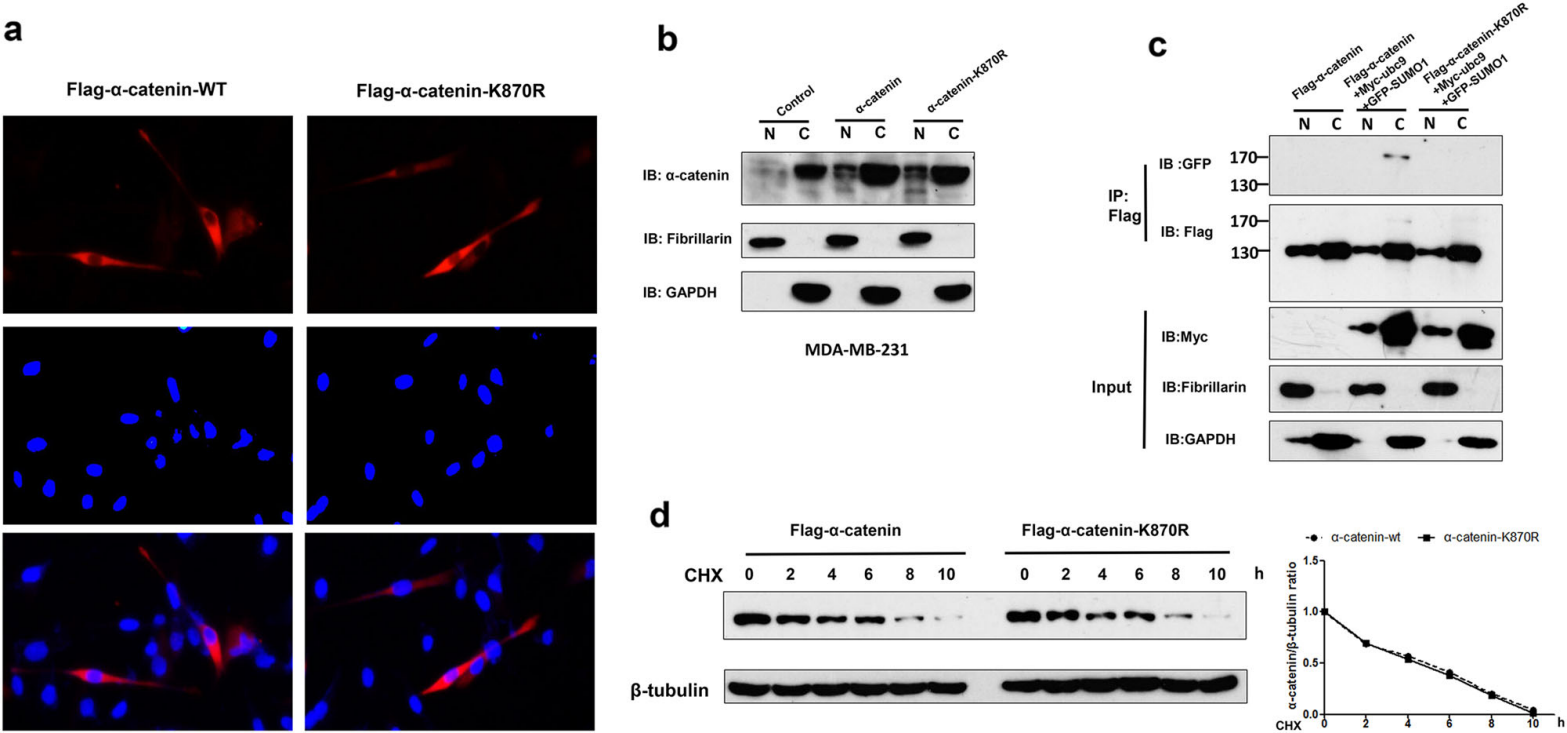
SUMOylation does not affect stability of α-catenin and cellular localization. **a** MDA-MB-231 cells transfected with indicated plasmids were stained with Flag antibody as previously described^52^. **b** MDA-MB-231 cells infected with indicated virus. Nuclear and cytoplasmic extraction were detected using western blot. Fibrillarin and GAPDH were measured to normalize protein abundance of α-catenin, respectively. **c** HEK293T cells were transfected with indicated plasmids. Cytosolic and nuclear fractions were inmmunoprecitated using Flag gel. Purified cell extracts were tested by western blot. **d** HEK293T cells transfected with WT Flag-α-catenin or K870R were treated with 50 mg ml^−1^ CHX, and cell lysates were detected using western blot

Since SUMOylation usually regulates substrate subcellular distribution, we wondered whether α-catenin SUMOylation is required for localizations to adherens junctions by transfecting WT Flag-α-catenin or the K870R mutant into MDA-MB-231 cells and monitoring α-catenin subcellular localization using immunofluorescent staining. The results showed that α-catenin mainly aggregated in the cytoplasm, regardless of mutation status (Fig. 3a). To confirm this phenomenon, we therefore detected the subcellular distribution of WT α-catenin or its SUMOylation mutant and observed the similar results when using a cell fractionation assay (Fig. 3b and Supplementary Figure 1). We then analyzed the subcellular distribution of α-catenin-SUMO. HEK293T cells were transfected with WT Flag-α-catenin or with K870R mutant Flag-α-catenin together with or without of Ubc9 and SUMO1 and analyzed α-catenin-SUMO localization. As expected, SUMOylation did not affect α-catenin nucleus and cytoplasm distribution (Fig. 3c). However, expected α-catenin-SUMO1 band was mainly evident in the cytoplasm (Fig. 3c), indicating that α-catenin-SUMO1 is distributed in the cytoplasm. Since SUMOylation usually affects target protein degradation rates, we therefore detected the protein stability of WT α-catenin and its SUMOylation mutant K870R. We observed that both WT α-catenin and the K870R α-catenin mutant were degraded at a similar rate (Fig. 3d). Together, SUMOylation does not affect α-catenin subcellular distribution and protein stability.

### α-catenin SUMOylation inhibits NF-κB pathway

**Fig. 4:**
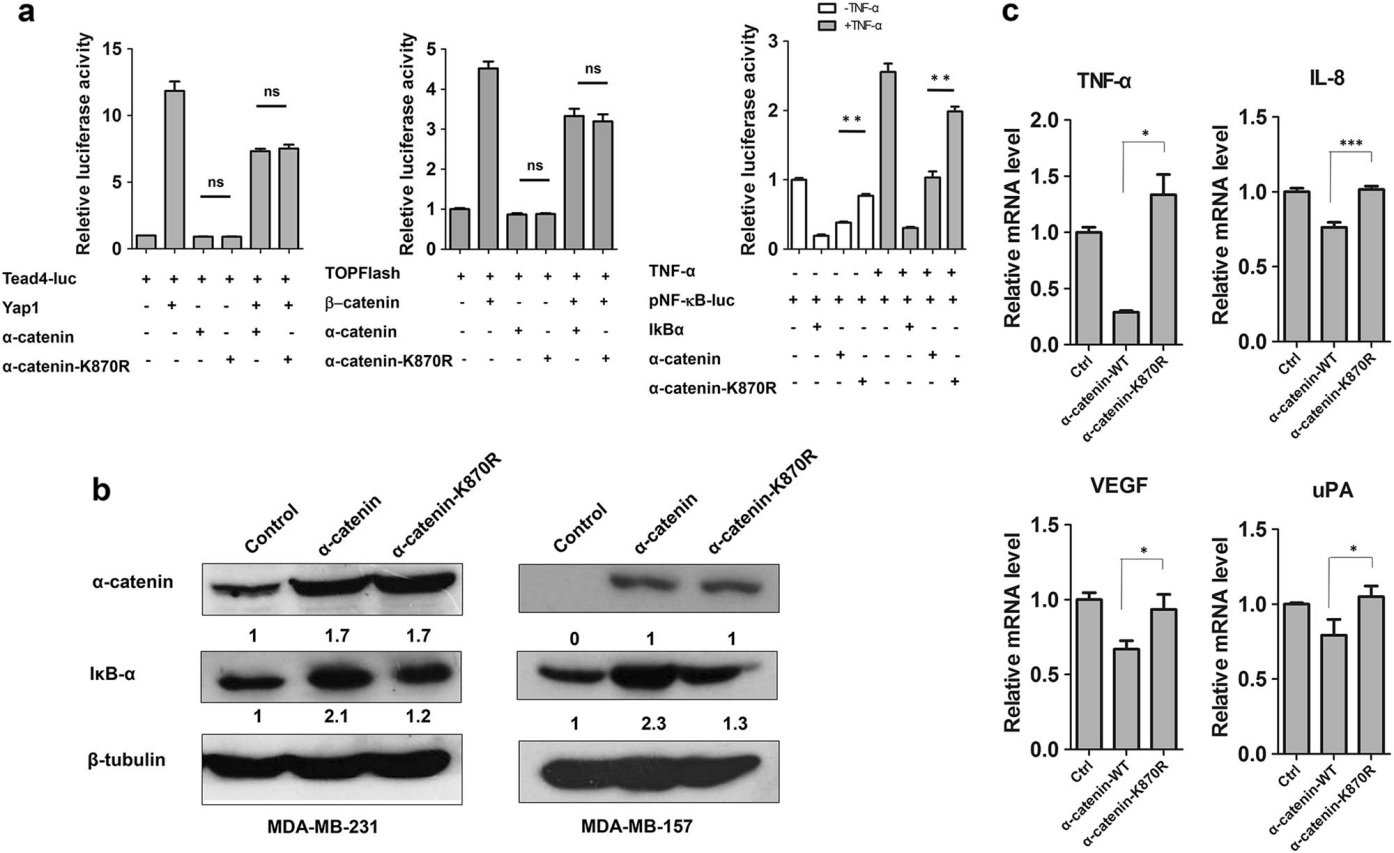
α-catenin SUMOylation inhibits NF-κB pathway. **a** HEK293T cells were transfected with indicated plasmids. Luciferase activity was measured with or without pre-treating of the cells with 10 ng ml^−1^ TNF-α 4 h (for pNFκB luciferase). **b** MDA-MB-231 and MDA-MB-157 cells transfected with wild-type α-catenin or its mutant K870R and then subjected to Western blot by anti-α-catenin or anti-IκBα antibody, respectively. **c** Real-time PCR was used to measure the mRNA levels of NFκB target genes *TNF-α*, *IL-8*, VEGF and *uPA* in MDA-MB-157 cells stable transfected with wild-type α-catenin or its mutant K870R. *n* = 3 wells per group

α-catenin is a tumor suppressor involved in Wnt^11,24^, Hippo-Yap^6,7^ and NF-κB signaling^9,10^ pathways. To determine whether SUMOylation of α-catenin regulates these pathways, we performed TOPFlash, Tead4 luciferase, and pNFκB-luciferase assays in HEK293T cells, respectively. Unexpectedly, the K870R α-catenin mutant showed significantly reduced inhibition of the pNF-κB-luciferase with or without TNF-α stimulation, whereas the α-catenin SUMOylation mutant did not affect TOPFlash and Tead4 luciferase activity compared to WT α-catenin (Fig. 4a). Since α-catenin inhibits NF-κB signaling by stabilizing IκBα in basal-like breast cancer^10^, we attempted to explore whether IκBα protein abundance is affected by α-catenin SUMOylation. For this hypothesis, we generated MDA-MB-231 and MDA-MB-157 cells that stably express WT α-catenin or the K870R α-catenin mutant using lentivirus. MDA-MB-157 cells carry a biallelic inactivating mutation in α-catenin and have therefore lost α-catenin expression^1^. α-catenin expression was comparable between the α-catenin mutant and WT when assessed using western blotting (Fig. 4b), indicating that the α-catenin K870R mutant does not affect α-catenin transcription or protein stability. In addition, the α-catenin K870R mutant had little effect on IκBα protein level compared to WT α-catenin (Fig. 4b). To further evaluate the effects of α-catenin SUMOylation at K870 on the transcriptional output of the NF-κB pathway, we measured the mRNA expression of NF-κB target genes *TNF-α*, *IL-8*, *VEGF*, and *uPA*^25–27^ in WT α-catenin or K870R α-catenin mutant-expressing MDA-MB-157 cells. As expected, upregulation of WT α-catenin significantly suppressed these NF-κB target genes. In contrast, the SUMOylation-deficient K870R α-catenin mutant was unable to inhibit expression of these genes in MDA-MB-157 cells. (Fig. 4c). Moreover, α-catenin SUMOylation mutant did not affect its inhibitory effect of Hippo-Yap target genes *CTGF* and *CRY61* in MDA-MB-157 cells (Supplementary Figure. 2a). Surprisingly, no matter WT α-catenin or α-catenin SUMOylation mutant did not inhibit Wnt target genes *CCND1* and *C-MYC* in MDA-MB-157 cells (Supplementary Figure. 2b). Taken together, NF-κB pathway is regulated by α-catenin SUMOylation.

### SUMOylation enhances the interaction between α-catenin and IκBα

**Fig. 5:**
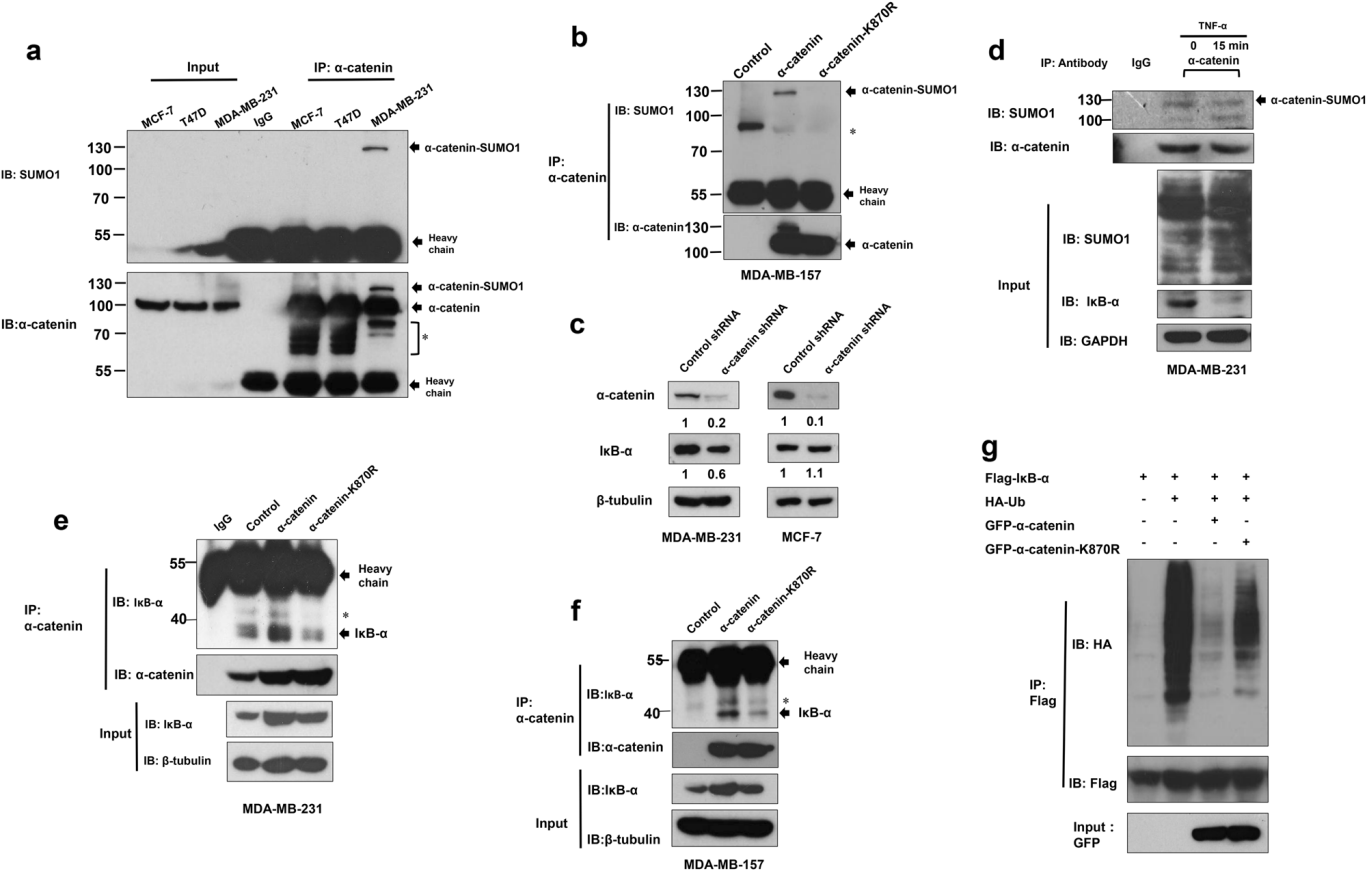
SUMOylation enhances α-catenin interacting with IκBα. **a** MCF-7, T47D, and MDA-MB-231 cell lysates were immunoprecipitated with anti-α-catenin antibody or control IgG followed by Western blot with anti-SUMO1 antibody. **b** MDA-MB-157 cells transfected with wild-type α-catenin or its mutant K870R and then subjected to inmmunoprecipitation with anti-α-catenin antibody followed by Western blot with anti SUMO1 antibody. **c** MCF-7 and MDA-MB-231 cells were transfected with α-catenin-shRNA or its control vector followed by Western blot with anti-α-catenin or anti-IκBα antibody, respectively. **d** MDA-MB-231 cells treated with or without 10 ng ml^−1^ TNF-α 15 min before lysis, and then lysates were immunoprecipitated with anti-α-catenin antibody or control IgG followed by Western blot with anti-SUMO1 antibody. **e** MDA-MB-231 cells transfected with wild-type α-catenin or its mutant K870R and then subjected to inmmunoprecipitation with anti-α-catenin antibody followed by Western blot with anti-IκB-α antibody. **f** In the same condition as **e**. **g** HEK293T cells were transfected with indicated plasmids. Cells were treated with 10 μM MG132 and 20 ng ml^−1^ of TNF-α for 30 min, and IκB-α was purified with anti-Flag gel followed by Western blot with HA or anti-Flag antibody. “*” non-specific bands

Since α-catenin SUMOylation deficiency attenuates its suppressive effect on the NF-κB pathway in MDA-MB-231 and MDA-MB-157 cells, we speculated that endogenous α-catenin was SUMOylated in these two cell lines. As expected, we found that endogenous α-catenin was modified by SUMO1 in MDA-MB-231 cells instead of MCF-7 and T47D cells (Fig. 5a), and the α-catenin-SUMO1 band was also present in lysates from MDA-MB-157 cells when cells were infected with WT α-catenin but not the K870R mutant (Fig. 5b), suggesting that endogenous α-catenin may mainly be SUMOylated in basal-like breast cancer cells. Since loss of α-catenin did not affect NF-κB pathway in the luminal subtype^10^, knockout of α-catenin in MCF-7 cells did not decrease protein level of IκBα (Fig. 5c). Moreover, the SUMOylation level of α-catenin was unaffected by TNF-α stimulation (Fig. 5d). Similar results were also observed when exogenous α-catenin SUMOylation assay was used in HEK293T cells (Supplementary Figure 3). Since substrate protein interactions usually affected by SUMOylation^28^. We therefore examined whether SUMOylation of α-catenin affects its ability to bind IκBα. Overexpression of WT α-catenin significantly increased the amount of IκBα co-immunoprecipitated. In contrast, overexpression of the K870R α-catenin mutant had no obvious effect on its interaction with IκBα in MDA-MB-231 cells (Fig. 5e). To further confirm that α-catenin SUMOylation increases its interaction with IκBα, an α-catenin-null MDA-MB-157 cell line was used. As expected, the amount of IκBα immunoprecipitated with WT α-catenin was much higher than with the K870R α-catenin mutant (Fig. 5f). To further confirm this, we examined IκBα ubiquitination. Overexpression of WT α-catenin significantly decreased the IκBα ubiquitination. However, K870R α-catenin mutant markedly lost this function (Fig. 5g). These results indicate that α-catenin SUMOylation increases its binding to IκBα.

### SUMOylation-deficient α-catenin attenuates its tumor-suppressor activity

**Fig. 6:**
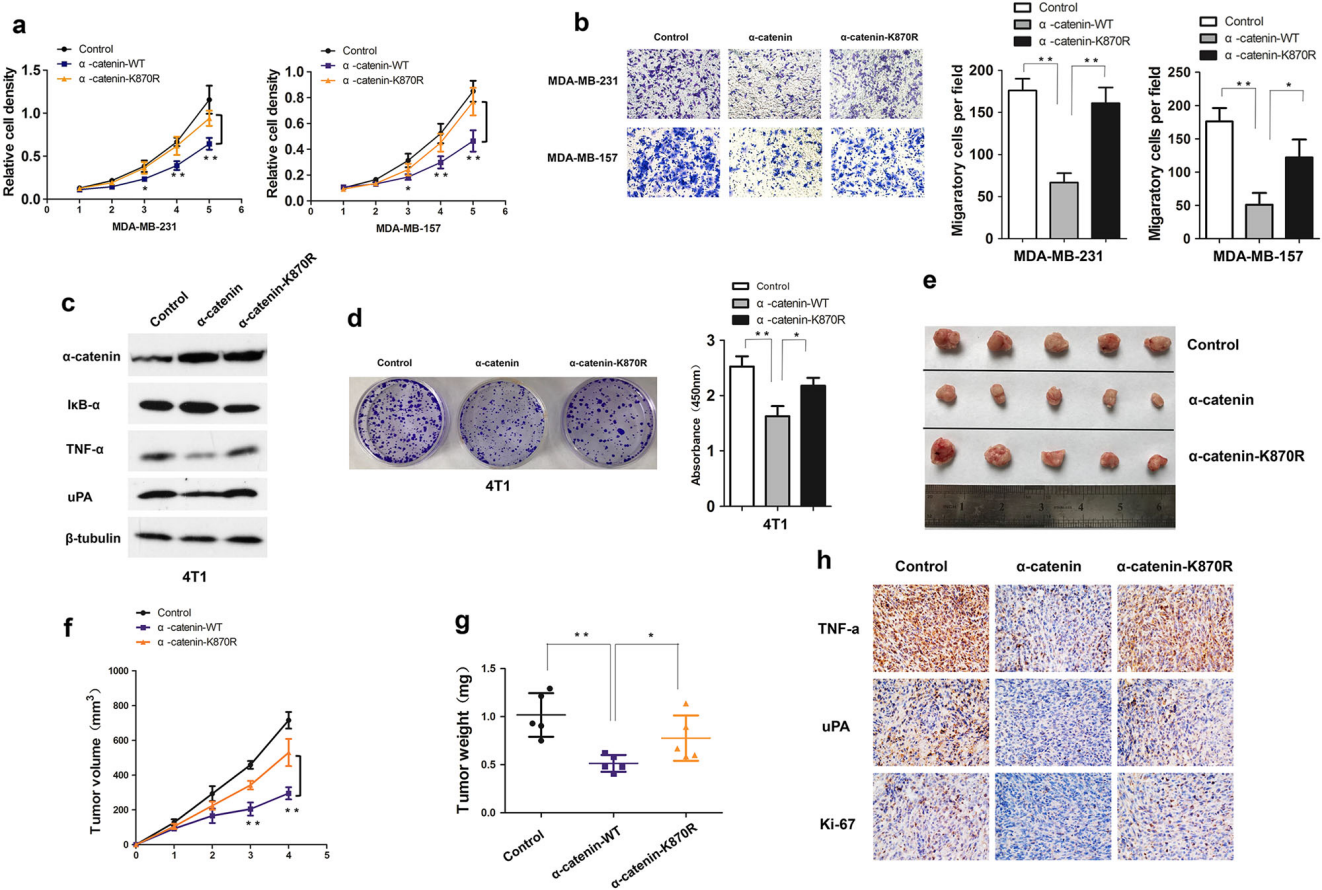
SUMOylation deficiency α-catenin attenuates its tumor-suppressor activity. **a** MTT assay was used to measured MDA-MB-231 and MDA-MB-157 cells proliferation ability. **b** Cell migration were performed in 24-well chambers. Cells (2 × 10^4^ per well) were transfected with control vector, wild-type α-catenin or its mutant K870R and then plated in the upper chamber. After 12 h of incubation, the migrating cells on the lower surface of the filter were stained and counted (left). The bar graphs show the number of migrating cells for each category of cells (right). *n* = 3 per well. **c** 4T1 cells were stably transfected with control vector, wild-type α-catenin or its mutant K870R and then subjected to Western blot with anti-α-catenin, anti-TNF-α, uPA, and anti-IκB-α antibodies. **d** 4T1 cells stably transfected with control vector, wild-type α-catenin or its mutant K870R were stained with crystal violet after 6 days of growth. *n* = 3 per well. **e** Tumor image. **f** Tumor growth by subcutaneously implanted 4T1 cells (5 × 10^5^ cells injected per mice) stably transfected control vector, wild-type α-catenin or its mutant K870R. **g** Tumor weight. *n* = 5 per group. **h** TNF-α, uPA, and Ki-67 immunohistochemical staining of subcutaneous tumors formed 4T1 cells transfected with Control alone, wild-type α-catenin or its mutant K870R, at 4 weeks after implantation (100×)

α-catenin has been shown to suppress the NF-κB pathway to inhibit cell proliferation and migration^9,10^ We explored whether α-catenin SUMOylation is responsible for tumor-suppressor function. In agreement with our hypothesis, WT α-catenin significantly inhibited cell proliferation (Fig. 6a and Supplementary Figure 4) and migration (Fig. 6b), whereas the K870R α-catenin mutants significantly lost the ability to suppress growth and migration. We then generated 4T1 cells to stably express the control empty vector, WT α-catenin or the K870R α-catenin mutant, and we analyzed NF-κB target genes TNF-α and uPA protein level. 4T1 cells is a rapidly growing and highly metastatic murine basal-like breast cancer cell line, in which the NF-κB signaling pathway is hyperactivated^29^. Like what we observed above, WT α-catenin significantly suppressed the protein level of TNF-α and uPA and inhibited colony formation. In contrast, K870R mutant lost these effects (Fig. 6c, d). We next subcutaneously transplanted stably transfected 4T1 cells expressing either α-catenin or K870R mutant in BABL/c mice. Both the WT and K870R mutant dramatically inhibited the subcutaneous growth of 4T1 cells. However, the tumor volumes and weights in the group with expression of the K870R α-catenin mutant were much greater than the group with expression of WT α-catenin (Fig. 6e–g). In addition, WT α-catenin 4T1 tumors exhibited downregulation of TNF-α, uPA and proliferation marker Ki-67. In contrast, the K870R α-catenin mutant significantly lost these abilities (Fig. 6h). Taken together, SUMOylation-deficient α-catenin has lost tumor-suppressor activity in vivo.

## Discussion

α-catenin is an important component of adherens junctions, which bind to actin filaments to maintain cellular connection integrity between neighboring cells^4,30^. Three α-catenin isoforms have been identified, including αE-catenin, αN-catenin and αT-catenin. αE-catenin is mainly expressed in epithelial tissues^31^ and is critical for the development of these tissues. For example, α-catenin-knockout nude mice display impaired hair growth and exhibit tumor-like nodules in skin tissues^9^. Accumulating evidence has revealed that downregulation or a loss of α-catenin expression contributes to multiple types of cancer, such as leukemia^2^, human skin squamous cell carcinoma^6–8^, colorectal^11,24,32–34^, breast^10^ and prostate cancer^35^. These studies and clinical correlation analyses have demonstrated that α-catenin inhibits cancer progression in different tissues via suppressing Wnt, Hippo-YAP, and NF-κB pathways, indicating that the function of α-catenin is affected by the surrounding environment. Numerous prior studies have focused on α-catenin biological function by manipulating protein levels using knockout or knock-in methods. However, the post-translational modifications of α-catenin need to be further elucidated. For example, a recent study has revealed that α-catenin is phosphorylated by casein kinase 1 and 2, which promotes intercellular adhesion^36^. Our data supports that a different kind of reversible post-translational modification may regulate α-catenin’s role in the NF-κB pathway. The SUMOylation site at lysine 870 that we identified in α-catenin is highly conserved, such as in the mouse, xenopus and zebrafish. We also find a similar SUMOylation site in αN-catenin and αT-catenin, indicating that these two isoforms may be SUMOylated in vivo.

Since no suitable therapy and drugs for triple-negative breast cancer cells (TNBC), it has a lower five-year life rate than other subtypes^37^. Emerging studies have identified that aberrant regulation of the SUMOylation pathway affects the progression of breast cancer. BRCA1 mutation status correlates with high Ubc9 expression which results in increased proliferation and migration of triple-negative breast cancer^38^. SUMOylation pathway also contributes to promoting the transition of breast cancer cells from a luminal to basal phenotype and is required to maintain cancer stem cell properties^39,40^. These studies may explain why endogenous α-catenin was not SUMOylated in MCF-7 and T47D (Fig. 5a). Activation of NF-κB, which is best known to be regulated by ubiquitination^41^, is tightly correlated with various kinds of tumors^29,42^. Degradation of IκBα is a key step for NF-κB activation, which subsequently activates downstream proinflammatory genes which initiate tumor cells proliferation and migration^43^. Like ubiquitination, NF-κB is also regulated by SUMOylation pathway^44^. For example, IκBα is modified by SUMO1, which prevents its ubiquitination by competing with Ubiquitin for the same lysine at position 21^17^. As it has been well-demonstrated that the SIM (SUMO-interacting motif) is important for mediating protein-protein interactions^45,46^, we speculate that IκBα contains a SIM motif which promotes the interaction between IκBα and SUMOylated α-catenin.

In this research, we demonstrate that α-catenin is SUMOylated at lysine 870, enhancing its interaction with IκBα, and therefore leading to upregulation of IκBα protein levels and inactivation of NF-κB pathway. We also demonstrate that α-catenin SUMOylation does not affect protein stability (Fig. 3d) and subcellular localization (Fig. 3a,b), which may explain why TOPFlash and Tead4 luciferase activities were unaffected by α-catenin SUMOylation (Fig. 4a). Furthermore, we demonstrate that α-catenin SUMOylation does not require TNF-α stimulation (Fig. 5d). These data provide a molecular mechanism for α-catenin SUMOylation and reveal an alternative to the canonical NF-κB signaling pathway that connects an important post-translational modification of α-catenin with the NF-κB signaling pathway. Collectively, our data has revealed that SUMO modification of α-catenin promotes its interaction with IκBα and allows for tumor suppressive activity.

## Materials and methods

### Plasmids and antibodies

Myc-labeled SUMO1, SUMO2, SUMO3, Flag-SENP1, Flag-SENP1m, GFP-SUMO1, and GFP-SUMO1-G97A were previously discribed^32,47,48^. Tead4 luciferase construct was a kind gift from Dr. Ximei Wu^49^. The following plasmids were a kind gift provided by Dr. Hai-long Piao: SFB-α-catenin construct, SFB IκBα construct, Myc-α-catenin construct, pLOC α-catenin construct, Myc-IκBα construct, HA-Ub (ubiquitin) construct, pNF-κB luciferase reporter construct, TOPFlash luciferase reporter construct, pGPIZ *CTNNA1* shRNA construct^10^. GFP-α-catenin was amplified from SFB α-catenin construct. GFP-α-catenin sense primer: 5′-CGGAATCCACTGCTGTCCATGCA-3′. GFP-α-catenin reverse primer: 5′-GGGGTACCCCTTAGATGCTGTCCATAGCTT-3′ pLOC α-catenin mutant (K870R), GFP α-catenin mutant (K870R) and Flag-α-catenin mutants (K87R, K870R) were generated using a site-directed mutagenesis kit (Stratagene, La Jolla, CA, USA).

The following antibodies were used: Flag, GFP, HA, Myc, α-catenin rabbit polyconal antibodies and Flag, GAPDH mouse monoclonal antibodies (Sigma, Saint Louis, MO, USA), rabbit anti-IκBα antibody (CST, 3 Trask Lane Danvers, MA, USA), rabbit SUMO1 polyclonal antibody (Abcam, Cambridge, MA, USA), SUMO1, Fibrillarin mouse monoclonal antibodies and rabbit anti-UBC9 (Santa Cruz Biotechnology, Dallas, Texas, USA), mouse anti-TNF-α, rabbit anti-uPA, rabbit anti-Ki-67 antibodies (Proteintech, Manchester, M3 3WF,UK), mouse anti-β-tubulin antibody (ZSGB-BIO, Beijing, China).

### Cell culture and transfection

MD-MBA-157 cells were purchased from China Infrastructure of Cell Line Resource and cultured under conditions specified by the provider. HEK293T, MCF-7, T47D, MD-MBA-231, and 4T1 cells were previously described^50^.Transfection assay was performed with Lipofectamine 2000 (Invitrogen, Auckland, New Zealand).

### Lentiviral transduction

Lentivirus containing medium was generated using three plasmids lentivirus package system. Cells were incubated with virus and selected using suitable antibiotic.

### Transwell assay

Transwell assay was previously described^51^. MDA-MB-231 cells (2 × 10^4^) or MDA-MB-157 cells (5 × 10^4^) were infected with virus and selected by blasticidin before plated.

### Luciferase reporter assay

Cells were plated into a 24-well plate at a confluency of 30%, and transfected with relevant plasmids 24 h later. The luciferase activity was tested as previously described^51^.

### Western blot, Co-immunoprecipitation, Immunohistochemistry

Cells extracts were generated using TNE lysis buffer. Western blot and Co-immunoprecipitation were previously described^32^. TNE lysis buffer (20 mM Tris-HCl [pH = 6.8], 100 mM NaCl, 1 mM EDTA, 0.5% NP-40, 10% Glycerol and complete protease inhibitor) containing 20 mM N-Ethylmaleimide (NEM, pierce) for exogenous SUMOylation assay. The method for analysis of endogenous α-catenin-SUMO1 was previously described^17^. Immunohistochemistry (IHC) were performed as previously described^51^.

### Cytoplasm and nucleus separation

Cytoplasm and nucleus separation assay was performed using Protein Extraction Kit (Wanleibio).

### Immunofluorescence staining

MDA-MB-231 cells were transfected with Flag-α-catenin or its SUMOylation mutant K870R using Lipofectamine 2000. Transfected cells were seeded at coverslip 48 h later. Immunofluorescence staining was described in our previously studies^32,51^.

### Cell growth assay

For crystal violet staining assay, cells were infected with virus containing medium, and then plated in 35 mm dish. After clones were clearly visible, cells were stained with crystal violet. MTT assay was described in our previous study^32^.

### Quantitative RT-PCR assays

Total RNA was extracted using RNAiso reagent (Takara Biotechnology, Dalian, China). cDNA was generated using a Reverse Transcription System (Takara). Normalized abundance of mRNA were measured using SYBR premix Ex Taq (Takara). The primers were designed as follows: TNF-α sense primer: cccaggcagtcagatcatcttc. TNF-α reverse primer: agctgcccctcagcttga. IL-8 sense primer: 5’-ttttgccaaggagtgctaaaga IL-8 reverse primer: aaccctctgcacccagttttc. VEGFA sense primer: cgggaaccagatctctcacc. VEGFA reverse primer aaaatggcgaatccaattcc. uPA sense primer: agtgtcagcagccccact. uPA reverse primer ccccctgagtctccctgg. CCND1 sense primer: gctgctcctggtgaacaagc. CCND1 reverse primer aagtgttcaatgaaatcgtgcg. C-MYC sense primer: cagctggagatgatgaccga. C-MYC reverse primer ttgatgaaggtctcgtcgtc. CTGF sense primer: gcccagacccaactatgattag. CTGF reverse primer: tctccgtacatcttcctgtagt. CRY61 sense primer: gactgtgaagatgcggttcc. CRY61 reverse primer ctgtagaagggaaacgctgc.

### Mice with homografts expressing WT α-catenin and K870R α-catenin

BALB/c mice (8 weeks old) were purchased from the Laboratory Animal Center of Dalian Medical University. Mice were managed and raised according to the procedures of the Ethics Committee for Biology and Medical Science of Dalian University of Technology. BALB/c mice were implanted subcutaneously with 4T1 cells infected with relevant virus. Tumor size and volume were recorded once a week. Tumor volume calculating method was used according to a previous study^10^.

### Statistical analysis

Data were showed as means ± SDs. An unpaired *t*-test was used when the results from two groups were compared. Experimental data is considered to be significant when *p *< 0.5. *p < 0.05, **p < 0.01.

## Electronic supplementary material

Supplementary figure legend

Supplementary Figure. 1

Supplementary Figure.2

Supplementary Figure.3

Supplementary Figure. 4
