# Supplementary material for: α-catenin SUMOylation increases IκBα stability and inhibits breast cancer progression
Source: Oncogenesis. 2018 Mar 13;7(3):28. doi: 10.1038/s41389-018-0037-7 (PMC5852976; doi:10.1038/s41389-018-0037-7)
Supplement: Supplementary file 1 — Supplementary figure legend [file 41389_2018_37_MOESM1_ESM.docx]

Supplementary Figure. 1

**SUMOylation does not affect stability of α-catenin and cellular localization.** MDA-MB-157 cells were transfected with wild-type α-catenin or its mutant K870R. Nuclear and cytoplasmic extraction were detected using western blot. Fibrillarin and GAPDH were measured to normalize protein abundance of α-catenin, respectively.

Supplementary Figure.2

**α-catenin SUMOylation inhibits NF-κB pathway** (**a**) and **(b)**the real-time PCR was used to measure the mRNA levels of Hippo-Yap and Wnt target genes *CTGF,* *CRY61*, *CCND1* and *C-MYC* in MDA-MB-157 cells stably transfected with WT α-catenin or its mutant K870R. n=3 wells per group.

Supplementary Figure. 3

**SUMOylation enhances α-catenin interacting with IκBα.** HEK293T cells co-transfected with Flag-α-catenin alone, Flag-α-catenin plus GFP-SUMO1 without or with TNF-α stimulation for 15 min, and cell lysates were inmmunoprecipited with flag gel and tested by western blot.

Supplementary Figure. 4

**SUMOylation deficiency α-catenin attenuates its tumor suppressor activity.**MDA-MB-231 and MDA-MB-157 cells stably transfected with control vector, wild-type α-catenin or its mutant K870R were stained with crystal violet after 6 (for MDA-MB-231 cells) or 9 days (for MDA-MB-157 cells) of growth. Stained clones were decolored with acetic acid and tested by 450nm. n=3 per well.
