# Supplementary figures and images for: α-catenin SUMOylation increases IκBα stability and inhibits breast cancer progression
Source: Oncogenesis. 2018 Mar 13;7(3):28. doi: 10.1038/s41389-018-0037-7 (PMC5852976; doi:10.1038/s41389-018-0037-7)

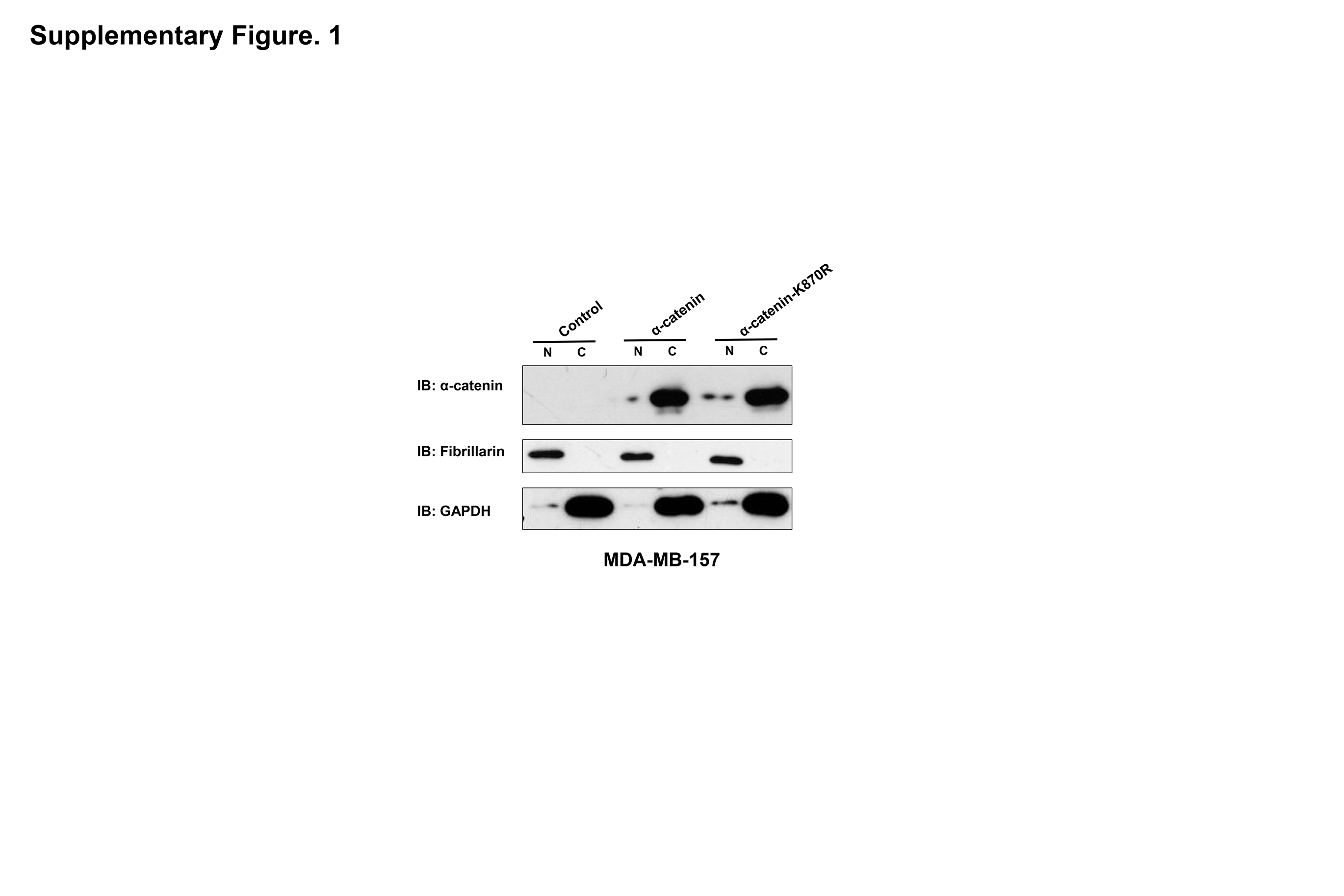

Supplement: Supplementary file 2 — Supplementary Figure. 1 [file 41389_2018_37_MOESM2_ESM.tif]

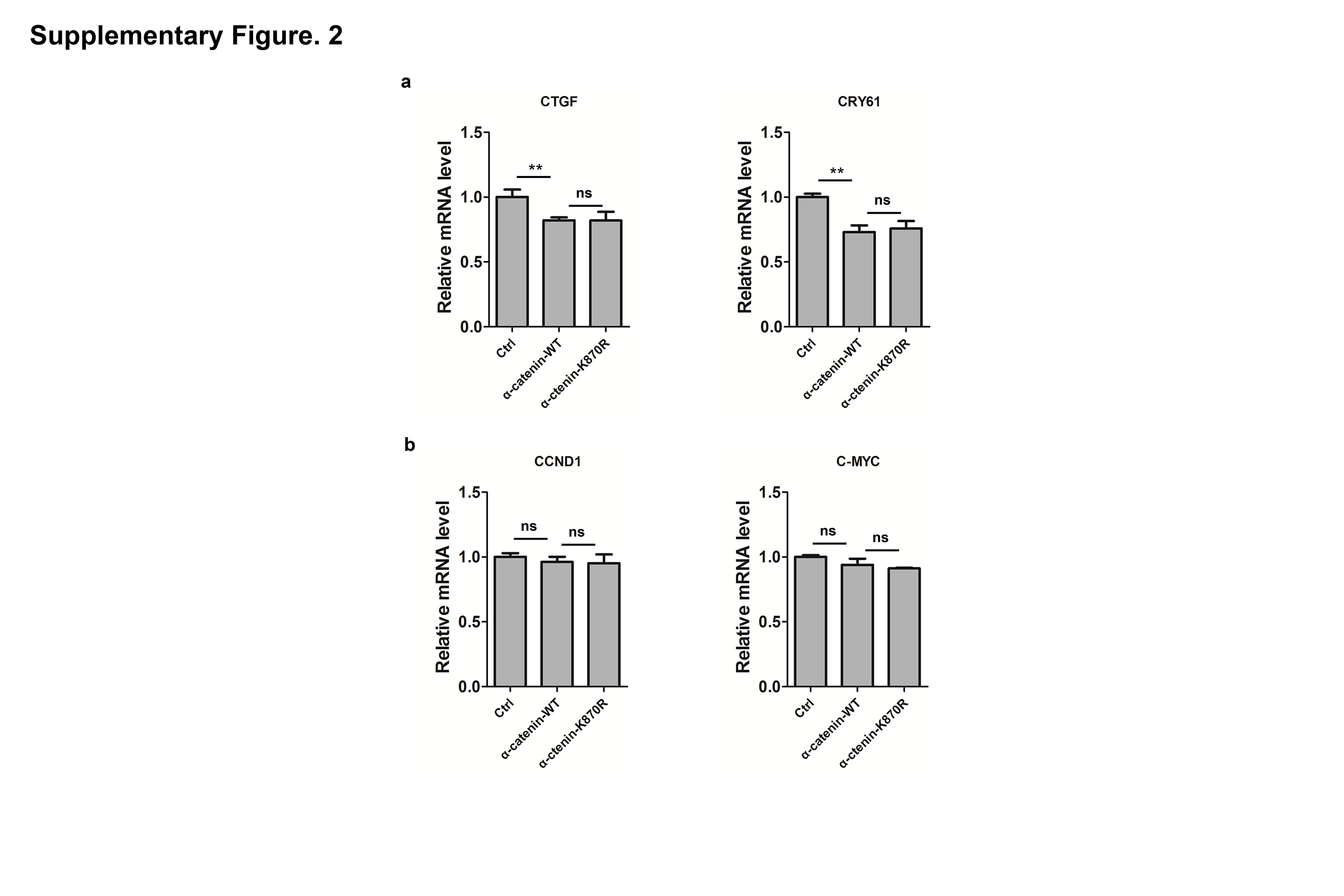

Supplement: Supplementary file 3 — Supplementary Figure.2 [file 41389_2018_37_MOESM3_ESM.tif]

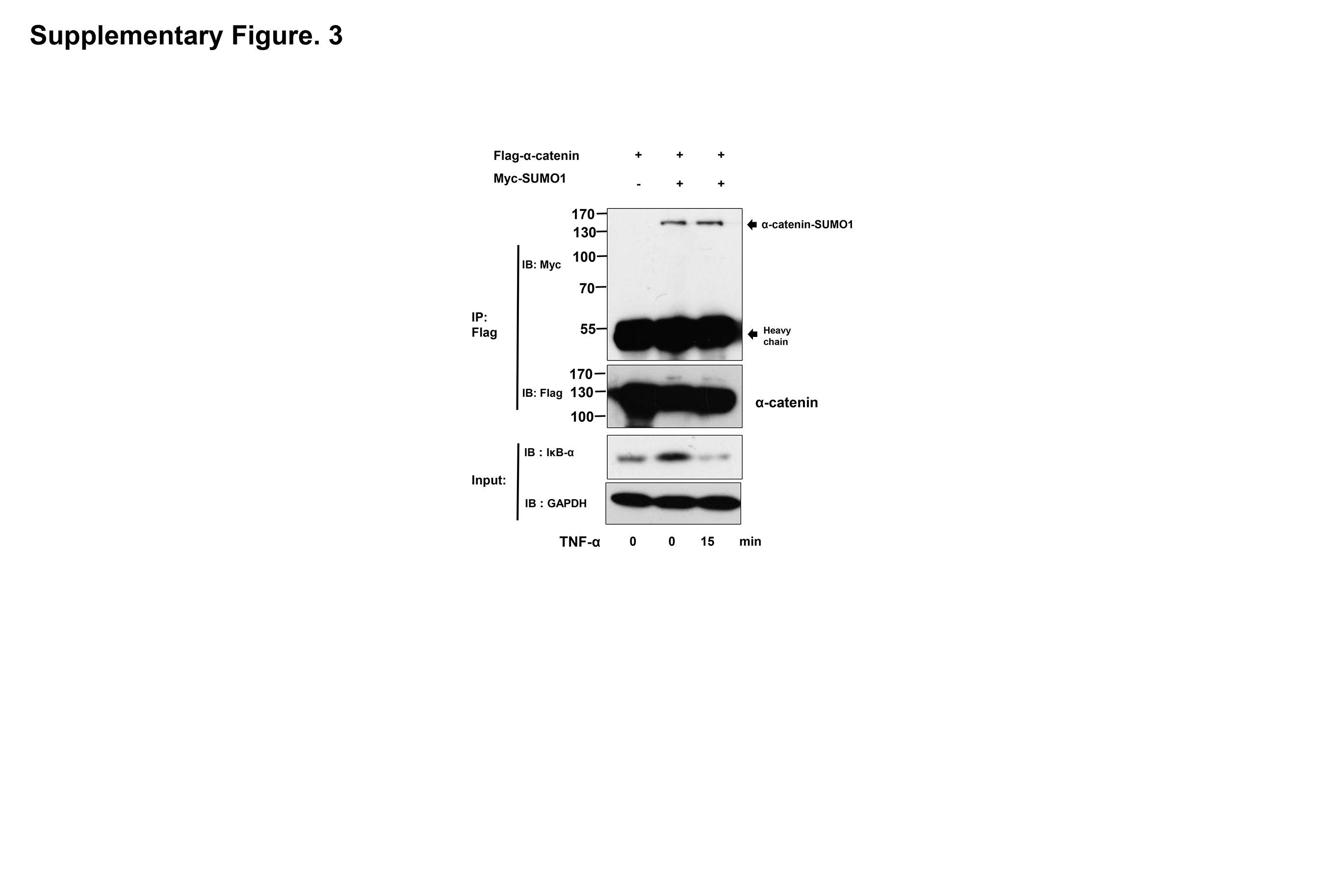

Supplement: Supplementary file 4 — Supplementary Figure.3 [file 41389_2018_37_MOESM4_ESM.tif]

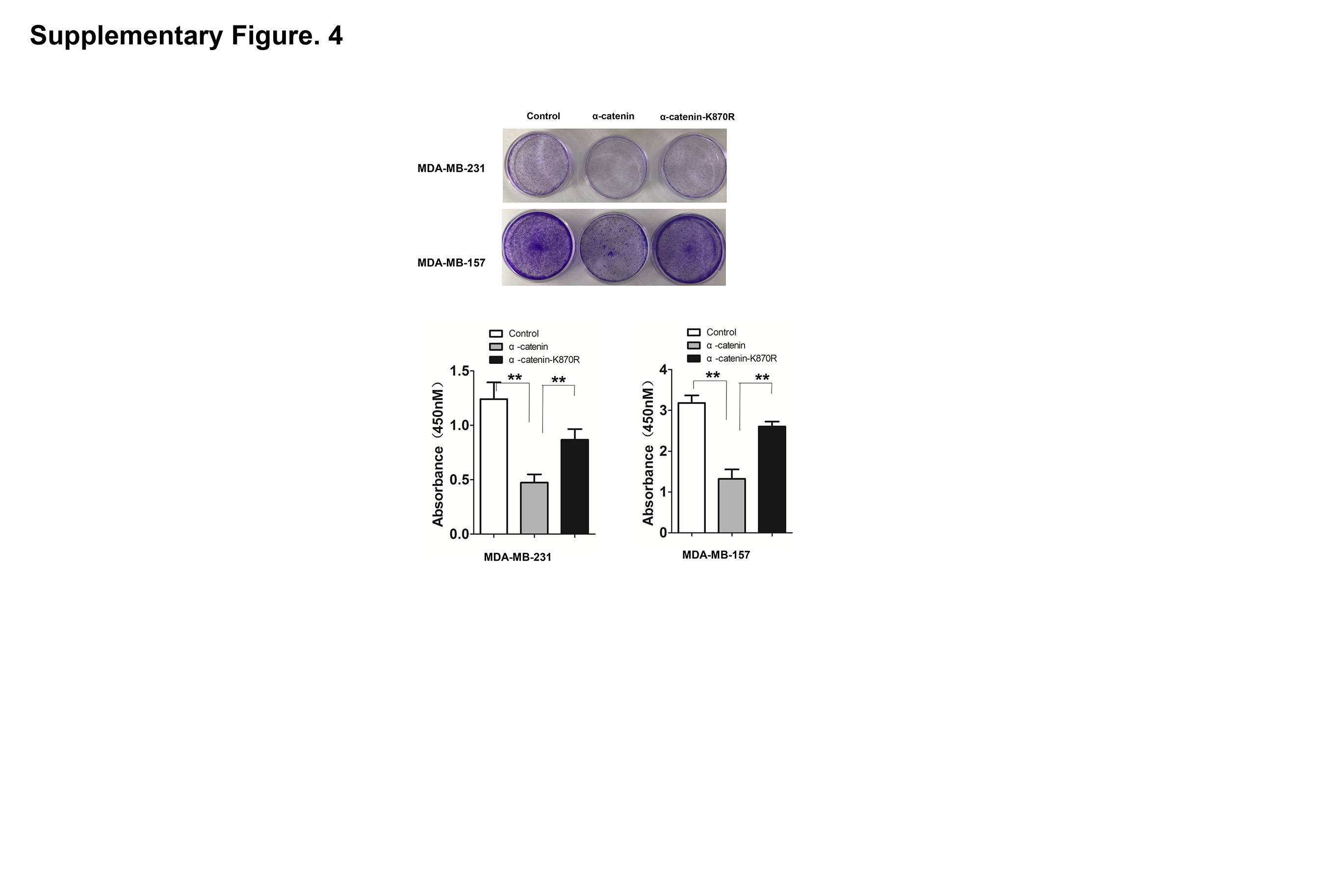

Supplement: Supplementary file 5 — Supplementary Figure. 4 [file 41389_2018_37_MOESM5_ESM.tif]
